# Validity and Reliability of a Fear of Failure Scale for Adolescents

**DOI:** 10.31083/AP39867

**Published:** 2025-04-21

**Authors:** Beliz Köroğlu, Feride Sülen Şahin Kıralp

**Affiliations:** ^1^Institute of Graduate Studies and Research, Guidance and Psychological Counselling, European University of Lefke, Lefke, Northern Cyprus, TR-10 Mersin, Turkey; ^2^Faculty of Education, Guidance and Psychological Counselling Department, European University of Lefke, Lefke, Northern Cyprus, TR-10 Mersin, Turkey

**Keywords:** fear of failure, adolescents, secondary school, reliability, validity

## Abstract

**Objectives:**

This study aimed to establish the validity and reliability of a fear of failure scale for adolescents.

**Methods:**

The study involved 279 secondary school students enrolled in the 2020–2021 academic year. Internal consistency, item-total score correlation, and split-half methods were used to determine reliability, while exploratory and confirmatory factor analyses were employed to determine validity.

**Results:**

The two split-in-half method calculations found a Guttman Split-in-Half coefficient of 0.855 and a Spearman-Brown coefficient of 0.857. Cronbach’s Alpha was 0.802 for the first half (items 1–9) and 0.774 for the second half (items 10–17). CFA analyses showed that a three-factor solution fit the data, but some goodness-of-fit indices fell below acceptable levels. To improve the model, error covariances of certain items were correlated based on modification indices. The final values were Minimum Discrepancy of Confirmatory Factor Analysis/Degrees of Freedom (CMIN/df) = 2.727, Goodness of Fit Index (GFI) = 0.911, Adjusted Goodness of Fit Index (AGFI) = 0.863, Comparative Fit Index (CFI) = 0.673, Root Mean Square Error of Approximation (RMSEA) = 0.079, χ^2^ = 160.9, Degrees of Freedom (DF) = 59. Factor loadings ranged from 0.52 to 0.83 for the first factor, 0.68 to 0.85 for the second factor, and 0.55 to 0.84 for the third factor.

**Conclusions:**

The fear of failure scale is a reliable and valid measurement tool. A review of the existing literature revealed a lack of scales that assess the physical, emotional, and thought dimensions of fear of failure among individuals aged 11–14 years. This gap underscores the potential for measurement-based research in this domain. Through this study, a valid and reliable scale was developed to evaluate fear of failure in adolescents within the 11–14 year-age range, thereby addressing this critical need.

## Main Points

1. It is important to have a scale that measures fear of failure among adolescent in order to arrange proper intervention programs in secondary schools.

2. There is not any valid and reliable Turkish scale to measure fear of failure in children between the ages of 11 and 14.

3. Because it consists of only 13 items, application and scoring of the scale is not time-consuming.

## 1. Introduction

Fear of failure is a phenomenon that occurs when the potential for success is evaluated (e.g., in education, sports, etc.). Fear of failure arises when an individual fears of not being able to adequately demonstrate his or her potential for success or of lacking the necessary skills [1, 2, 3]. On the other hand, fear of failure is explained as a type of avoidance related to performance or success [4]. It can also be defined as a negative feeling that emerges as a result of an individual’s expectation of the possibility of failure [2]. It is reported that individuals who associate failure with negative outcomes in particular accept failure as a threat and experience intense fear, resulting in burnout, as well as the endangerment of his/her mental and physical health and moral development [5].

Studies on the cognitive correlates of fear of failure suggest that it is a response to some kind of perceived threat or the consequences of not being able to achieve set goals [6]. The theory developed by McClelland* et al*. [7] suggested that people act with the motivation to accomplish things or avoid failure. The motivation to avoid failure basically involves evading shame and embarrassment [8]. On the other hand, people think that when they fail, they suffer from some consequences, such as lowered or lost self-worth, uncertainty, losing the interest of important people in their life, or disappointing or upsetting them [9]. The greater the individual’s belief that they may experience unpleasant outcomes, the greater the potential of fear of failure [10].

Certain societies or families are characterized with extremely high expectation of success. Academic success is a phenomenon that has gained global currency, guiding educational policies and leading initiatives to maximize it [11]. The academic success of their children is essential for parents, especially when it comes to their future careers. Burka and Yuen [12] reported that some Western societies in particular are achievement-oriented and therefore tend to produce extensive fear of failure. Gartenhaus [13] reported that individuals who feel pressurized for academic success are more likely to suffer from fear of failure.

As a minority group of, Turkish Cypriot youth face many difficulties in their education process due to embargoes imposed on the Turkish Republic of Northern Cyprus (TRNC) and the lack of international recognition, such as economic challenges, job opportunities, etc. As a result, they may experience higher levels of anxiety, perceive themselves as more inadequate when compared to their peers around the world, and struggle more to cope with failure.

Along with the presence of economic difficulties, the fact that Turkish Cypriots are not citizens of the European Union further increases the cost of education abroad. In addition to the mentioned economic reasons, other factors such as the fact that no country except Turkey, recognizes the passport of the TRNC, the rate of students studying abroad decreases every year because students face legal barriers such as visa procedures. According to the 2020–2021 statistics of the Higher Education Department of the Ministry of National Education of the TRNC, 68.85% of high school graduates continue their university education. Among them, 9% study in Turkey, 5.5% study in third countries, and 54.20% study in universities in the TRNC. Thus, the rate of students continuing their higher education outside of the TRNC is 14.64%. Five years ago, in the 2015-16 academic year, the rate of university students studying in Turkey, was 33.86%, in third countries was 10.43%, and in the TRNC was 55.7 percent. Looking at these figures, it is evident that the percentage of young Turkish Cypriots studying abroad is decreasing [14].

One of the primary reasons for the decline in the number of students studying abroad could be their status as part of a relatively small community. However, the challenges encountered by these children and young people often differ significantly from those faced by the majority. Factors such as family relationships, job opportunities, the geographic location of the region, peer relationships, quality of education, and economic level can be considered influential in this regard [15]. The literature implies that minority students who perceive themselves as such are inevitably destined to fail in this community because they belong to a minority group. Therefore, most minority students suffer from low motivation and poor success rates within the education system due to this perception [16]. When constraints related to accessing high-quality education and employment opportunities in rural areas are combined with family structure and geographic isolation, it means diminished aspirations for education among young individuals [17].

In addition, young students demonstrate a preference for staying in their hometowns due to strong familial ties and tight-knit community relationships. Paradoxically, they may also want to leave in the pursuit of better education and job opportunities [18]. Similarly, speaking for the TRNC case, students may feel pressurized by their parents to succeed, whereas they may also experience societal pressure that their parents face due to close family and kinship relationships.

Specifically, in the context of the TRNC, migration requires engaging in competition with other students benefiting from better educational standards in metropolitan regions with many job opportunities. Conversely, staying at home entails competing for highly limited employment opportunities. As a result of this system, due to the very limited opportunities available to young Turkish Cypriots, they inevitably feel more anxious and inadequate compared to their peers around the world. In their educational lives, young Turkish Cypriots experience higher levels of anxiety, believing that only highly successful individuals can pursue education abroad. This leads them into a vicious cycle of anxiety and feelings of failure.

It is frequently reported that fear of failure is mostly seen during adolescence, when personal or social expectations are at the highest level [19, 20, 21, 22, 23]. However, the lack of an instrument that directly measures this fear, which is frequently encountered in academic life, especially in adolescence, increases the importance of this research. As a result of the literature review, it is seen that the article titled “The Performance Failure Appraisal Inventory”, which stands out among the studies measuring fear of failure, has conducted reliability and validity studies in different languages and cultures [2]. However, this inventory measures only the cognitive aspects of failure appraisal [10, 24, 25, 26]. According to the results of the extended literature review, it is seen that some scales that can only be applied to people aged 18 and over which measure fear of failure by associating it with some variables are used in Turkish sources. The review displayed that no scale measures the physical, emotional, and cognitive dimensions of fear of failure in the Turkish language [27, 28, 29, 30, 31]. On the other hand, only Kandemir’s primary data study for university and high school students was detected in the Turkish literature as a useful tool. This scale was chosen because it includes items that cover the physical, thoughts, and emotional dimensions of fear of failure [32].

Accordingly, it was decided that it would be useful to conduct a reliability and validity study for this age group to measure various aspects fear of failure. After obtaining the necessary permissions and information from the developer of the scale for this research, a validity and reliability study was conducted. It is believed that this study will shed light on the deeper perceptions underlying the fear of failure in school among children who grow up in small communities or minority groups. This study, conducted on a minority group, is also considered to provide a scale that school guidance services and school counselors could utilize.

## 2. Material and Method

### 2.1 Sample and Collection of Data

The population of the study was determined as secondary school students in Turkish Republic of Northern Cyprus. The sample of the research consisted of the students enrolled in 2021–2022 academic year who were identified using convenience sample method. The study was conducted in accordance with the Declaration of Helsinki, and the protocol was approved by the Ethics Committee of European University of Lefke (approval number: BAYEK019.08, Date 19.01.23). The researcher facilitated the distribution of surveys to students’ parents by contacting personnel in the guidance services of schools. Students whose parents gave their consent for participation in this study accessed the surveys via a Google Form link. The introductory section of the survey was prepared with full consideration of all ethical principles. In accordance with these principles, students were provided with an informed consent form detailing the purpose of the research, data confidentiality, the voluntary nature of student participation, and the assurance of anonymity.

In this research, the sample size was determined by considering the following prerequisites regarding the relationship between sample size and the number of items:

-The sample size should be larger than the number of variables.

-The sample size should be at least 50.

-The observation-to-item ratio should be at least 5:1 [33, 34].

There are different opinions about sample size in scale reliability studies. Accordingly, it is argued that a sample size of 2 to 10 times the number of items in the scale should be achieved [35, 36, 37]. Therefore, it was calculated that reaching 34–170 participants would be sufficient for the reliability of the Fear of Failure Scale consisting of 17 items.

Data were collected from two different samples for the validity and reliability studies of the scale. A total of 283 volunteer students were reached, but the questionnaire forms of 4 students were not included in the statistical analysis because they answered the questions randomly and left too many questions unanswered.

Different data sets were used for exploratory factor analysis (EFA) and confirmatory factor analysis (CFA). EFA was conducted with 75 students out of 279 students who suffered from high level of fear of failure, and CFA was conducted with the remaining 204 students. Among the 75 students subjected to EFA, 58.2% were female and 41.8% were male. The mean age of the participants was calculated as 12.76 ± 0.70. Among the 204 students subjected to CFA, 56.4% were female and 43.6% were male. The mean age of the sample was calculated as 12.8 ± 0.85. The gender and age distributions in the data sets collected for EFA and CFA were similar.

### 2.2 Measurement Instruments

#### 2.2.1 Personal Information Form

Socio-demographic variables of the participants were determined in the personal information form prepared by the researcher. In the personal information form, students were asked questions such as gender, age, grade, number of siblings, rank of birth among their siblings, education level of their parents, perceived economic status, perceived parental attitude, perceived academic success, place of residence, factors that encourage them to study, and how they spend their extra-school time.

#### 2.2.2 Fear of Failure Scale

The Fear of Failure Scale was developed by Kandemir in 2012 who [32], after a comprehensive review of the literature on fear of failure, developed an item pool related to this construct. This item pool was subsequently submitted to relevant experts for evaluation. Based on the feedback received, Kandemir [32] finalized the Fear of Failure Scale, consisting of 17 items. In his research titled “Explanation of Students’ Academic Procrastination Behaviors with Anxiety, Fear of Failure, Self-Esteem and Achievement Goals”, Kandemir [32] employed primary data (collected from 315 final-year secondary school students) to assess the psychometric properties of the Fear of Failure Scale. The results of the factor analysis suggested a unidimensional structure, with factor loadings ranging from 0.41 to 0.76, and the Cronbach’s alpha coefficient for internal consistency calculated as 0.82. It is important to note that this scale, while developed and utilized in Kandemir’s study [32], has not been published in a peer-reviewed journal. The author allows the scale to be used in subsequent studies, on the condition that researchers conduct new validity and reliability analyses and report their findings. Furthermore, in 2019, Kandemir and Hayran [29] conducted a validity and reliability study of the Fear of Failure Scale for university students. Their analysis confirmed the unidimensional structure of the 17-item scale, with a Cronbach’s alpha coefficient of 0.91, indicating high internal consistency.

In the present study, with permission from Kandemir [32], the scale items were reviewed by three educational science experts to evaluate their appropriateness for a sample of middle school students in TRNC. The scale was administered based on the experts’ feedback, which confirmed the suitability of the items.

#### 2.2.3 Scale for Social Anxiety in Adolescents 

The social anxiety scale for adolescents was first developed by La Greca 1999 [38]. In 2007, Aydın and Sütçü [39] conducted psychometric studies for Turkish adolescents and adapted this scale to Turkish. The “Social Anxiety Scale for Adolescents” (SAS-A) is a scale consisting of 18 items and three factors that aims to assess the social anxiety level of secondary school students. The sub-dimensions of the scale are Fear of Negative Evaluation (FNE), Social Avoidance and Restlessness in General Situations (SAD-G), and Social Avoidance and Restlessness in New Situations (SAD-N). The Social Anxiety Scale for Adolescents is a five-point Likert-type rating scale. The answer “never” is given 1 point, and the answer “always” is given 5 points [39] Cronbach’s Alpha internal consistency coefficient for the entire adolescent social anxiety scale was found to be 0.88, and the coefficient for split-in-half method reliability was found as 0.85.

## 3. Results

### 3.1 Reliability of the Scale

Within the scope of the reliability analysis of the Fear of Failure Scale, the split-in-half method reliability was examined in order to determine the internal consistency coefficient, item total score correlations, and consistency between the responses obtained from the scale. As part of the reliability analysis of the Fear of Failure Scale, the internal consistency coefficient, item-total score correlations, and finally, split-half reliability were examined to assess the consistency of responses obtained from the scale.

The internal consistency coefficient of the 17-item Fear of Failure scale was calculated as 0.880. According to the item analysis conducted to determine the total score predictive power and discrimination of the scale items, it was found that the item-total score correlations varied between 0.326 and 0.679. An item-total score correlation below 0.30 indicates that the item is not adequate to distinguish the feature to be measured [40]. In this study, no total correlation score was calculated below the specified value; therefore, it was determined that the total score predictive power and discrimination of the scale items were satisfactory (Table 1). Another technique used was the split-half method. Spearman-Brown, Guttman Split-in-Half and Cronbach’s Alpha values were calculated to determine the split-in-half method reliability of the scale. According to these calculations, the Guttman Split-in-Half coefficient was found to be 0.855, and the Spearman-Brown coefficient was found as 0.857. Cronbach’s Alpha value was calculated as 0.802 for the first half (items 1–9) and 0.774 for the second half (items 10–17) of the scale [41].

**Table 1.** **Item analysis results**.

Items	Scale mean score when item is removed	Scale variance when item is removed	Corrected total item correlation	Scale alpha value when item is removed
1. Getting low grades on exams has always scared me.	30.97	96.837	0.456	0.873
2. Failure is the worst situation a student can experience.	30.96	94.742	0.544	0.870
3. Failure is a major fear for me.	31.13	95.225	0.546	0.869
4. I am not able to study for exams that I think I may fail.	31.93	100.712	0.350	0.877
5. I sometimes have problems with people around me due to my fear of failure.	31.87	98.090	0.542	0.870
6. Failure is enough to lose the favor of others.	31.59	93.759	0.650	0.865
7. I think I will not be a very successful person in the future, due to my fears.	31.49	99.659	0.330	0.878
8. Homeworks or exams scare me more when compared to other people.	31.68	94.599	0.628	0.866
9. I am not able to start studying because I am afraid of failure.	32.05	97.240	0.642	0.868
10. Sleep evades me if I think I will fail my exams.	31.68	100.869	0.326	0.878
11. The thought of failure affects my academic performance.	31.53	95.739	0.588	0.868
12. When I prepare for an exam, the first thing that comes to my mind is failure.	31.45	95.819	0.442	0.875
13. I sometimes cannot concentrate on my classes due to fear of failure.	31.65	98.257	0.434	0.874
14. Failure is not a situation that can be explained.	31.65	92.338	0.679	0.864
15. I feel nervous and tense before difficult exams.	31.23	95.772	0.515	0.871
16. Receiving a low grade affects me for a prolonged period of time.	31.43	98.329	0.397	0.876
17. I sometimes lose my self-confidence due to fear of failure.	31.59	93.003	0.662	0.865

### 3.2 Validity of the Scale 

Kaiser-Meyer-Olkin Test and Bartlett Tests were performed to determine the fitness of the scale for factor analysis before performing EFA to identify the validity of the Fear of Failure Scale. Kaiser-Meyer-Olkin Test was calculated as 0.785, and Bartlett Test was calculated as χ^2^(df): 585.069(136), *p*: 0.001. The Kaiser-Meyer-Olkin Test result being higher than 0.60 and the Bartlett Test result being significant indicates that the scale is fit for factor analysis [41].

According to the results of the Principal Component Analysis, it was determined that the scale had a 5-factor structure with an eigenvalue above 1 explaining 70.30% of the total variance (Table 2).

**Table 2.** **Number of factors and percentage of variance explained based on eigenvalue statistics**.

	Initial	Eigenvalues		Loading value	Variance of squares %	Total
Component	Total	Variance %	Cumulative %	Total	Variance %	Cumulative %
1. Getting low grades on exams has always scared me.	6.032	35.485	35.485	6.032	35.485	35.485
2. Failure is the worst situation a student can experience.	2.125	12.499	47.984	2.125	12.499	47.984
3. Failure is a major fear for me.	1.456	8.563	56.547	1.456	8.563	56.547
4. I am not able to study for exams that I think I may fail.	1.235	7.264	63.811	1.235	7.264	63.811
5. I sometimes have problems with people around me due to my fear of failure.	1.103	6.489	70.300	1.103	6.489	70.300
6. Failure is enough to lose the favor of others.	0.875	5.148	75.449			
7. I think I will not be a very successful person in the future, due to my fears.	0.712	4.186	79.635			
8. Homeworks or exams scare me more when compared to other people.	0.594	3.495	83.129			
9. I am not able to start studying because I am afraid of failure.	0.504	2.963	86.093			
10. Sleep evades me if I think I will fail my exams.	0.468	2.751	88.843			
11. The thought of failure affects my academic performance.	0.363	2.134	90.978			
12. When I prepare for an exam, the first thing that comes to my mind is failure.	0.341	2.009	92.986			
13. I sometimes cannot concentrate on my classes due to fear of failure.	0.312	1.838	94.824			
14. Failure is not a situation that can be explained.	0.274	1.613	96.437			
15. I feel nervous and tense before difficult exams.	0.252	1.482	97.919			
16. Receiving a low grade affects me for a prolonged period of time.	0.219	1.291	99.210			
17. I sometimes lose my self-confidence due to fear of failure.	0.134	0.790	100.000			

The original form of the scale was single-factor, which was first developed by Kandemir (2012) [32] for high school students preparing for the university exam. The validity and reliability study conducted by Kandemir and Hayran [29] for university students in 2019 also showed single-factor structure. In this study, five factors were initially found; however, since the original scale by Kandemir and Hayran [29] was also single-factor, the Velicer MAP test was additionally used. The Velicer Minimum Average Partial Correlation (MAP) Test was applied to determine the factor structure of the scale. Several researchers report that the Velicer MAP Test is the method that best reveals the true factor structure [42, 43]. As a result of the analysis conducted using the Velicer MAP test, it was determined that the Fear of Failure Scale had a 3-factor structure. As can be seen in Table 3, the smallest partial correlation mean square and the smallest partial correlation 4th power mean are 0.0362 and 0.0035, respectively. According to Velicer’s original formula (1976) [43], the MAP test identified a two-factor structure, while the revised formula (2000) [44] indicated a three-factor structure (Table 3). In light of the Velicer MAP Test findings, EFA was repeated as a three-factor structure. According to this analysis, the 3-factor structure with an eigenvalue above 1 explaining 56.55% of the variance is presented in Table 4. The scale items were divided into sub-dimensions with the Direct Oblimin rotation process. As a result of this analysis, the first sub-dimension consisting of 6 items explaining 35.485% of the variance (Eigenvalue = 6.032), the second sub-dimension consisting of 8 items explaining 12.499% of the variance (Eigenvalue: 2.125), and the third sub-dimension consisting of 3 items explaining 8.563% of the variance (Eigenvalue: 1.456) were formed. As a result of EFA, items 5, 6, 9, 12, 13, and 17 constitute the first sub-dimension, items 1, 2, 3, 4, 7, 8, 11, and 14 constitute the second sub-dimension, and items 10, 15, and 16 constitute the third sub-dimension (Table 4).

**Table 3.** **Velicer MAP test for fear of failure scale**.

Number of factors	Partial correlation mean square	4th power mean score of partial correlations
0.0000	0.1155	0.0230
1.0000	0.0401	0.0037
2.0000	0.0362	0.0036
3.0000	0.0379	0.0035
4.0000	0.0431	0.0047
5.0000	0.0458	0.0059
6.0000	0.0510	0.0066
7.0000	0.0589	0.0106
8.0000	0.0669	0.0128
9.0000	0.0868	0.0163
10.0000	0.1028	0.0259
11.0000	0.1313	0.0396
12.0000	0.1604	0.0579
13.0000	0.2183	0.1010
14.0000	0.3122	0.1821
15.0000	0.4856	0.3550
16.0000	1.0000	1.0000

MAP, Minimum Average Partial.

**Table 4.** **Exploratory factor analysis results**.

Question No	Questions	Factor 1	Factor 2	Factor 3
13	I sometimes cannot concentrate on my classes due to fear of failure.	0.863		
5	I sometimes have problems with people around me due to my fear of failure.	0.758		
17	I sometimes lose my self-confidence due to fear of failure.	0.684		
12	When I prepare for an exam, the first thing that comes to my mind is failure.	0.682		
6	Failure is enough to lose the favor of others.	0.612		
9	I am not able to start studying because I am afraid of failure.	0.590		
2	Failure is the worst situation a student can experience.		0.788	
3	Failure is a major fear for me.		0.756	
11	The thought of failure affects my academic performance.		0.690	
8	Homeworks or exams scare me more when compared to other people.		0.678	
14	Failure is not a situation that can be explained.		0.643	
1	Getting low grades on exams has always scared me.		0.618	
4	I am not able to study for exams that I think I may fail.		0.411	
7	I think I will not be a very successful person in the future, due to my fears.		0.374	
16	Receiving a low grade affects me for a prolonged period of time.			0.750
15	I feel nervous and tense before difficult exams.			0.716
10	Sleep evades me if I think I will fail my exams.			0.677
Variance Explained %		35.485	12.499	8.563
Eigenvalue		6.032	2.125	1.456
Cronbach Alpha		0.843	0.814	0.715

Upon a detailed examination of the items based on the identified factors, it was observed that the first factor consists of negative thought patterns. This subdimension, associated with negative cognitive schemas, includes thoughts that the student may be perceived as inadequate by those around them, that they will not be able to focus on classes or exams due to fear, and that they will fail because they are unable to begin studying. The items related to the second factor were found to be predominantly associated with the individual’s negative emotions. In this dimension, statements reflecting fear and intense anxiety were most frequently observed. Finally, the items under the third factor were determined to pertain to physical manifestations of fear, such as sleep disturbances, tension, and restlessness.

CFA was performed using AMOS 24 to test the goodness of fit of the structure obtained from EFA. As mentioned before, data was collected from a different sample for this analysis. Examination of the goodness of fit indices showed that the factor structure could be defined with a three-factor solution. However, since some of the values obtained were below acceptable limits, error covariances of certain items were correlated in line with the recommendations of the correction indices. However, according to the item analysis results, the estimate values of items 4, 7, 11 and 14 were below 0.70, as a result of which these items were removed from the scale. The following calculations were made before removing these four items: Minimum Discrepancy of Confirmatory Factor Analysis/Degrees of Freedom (CMIN/df) = 3.159, Goodness of Fit Index (GFI) = 0.845, Adjusted Goodness of Fit Index (AGFI) = 0.795, Comparative Fit Index (CFI) = 0.380, and Root Mean Square Error of Approximation (RMSEA) = 0.88.

**Fig. 1.**
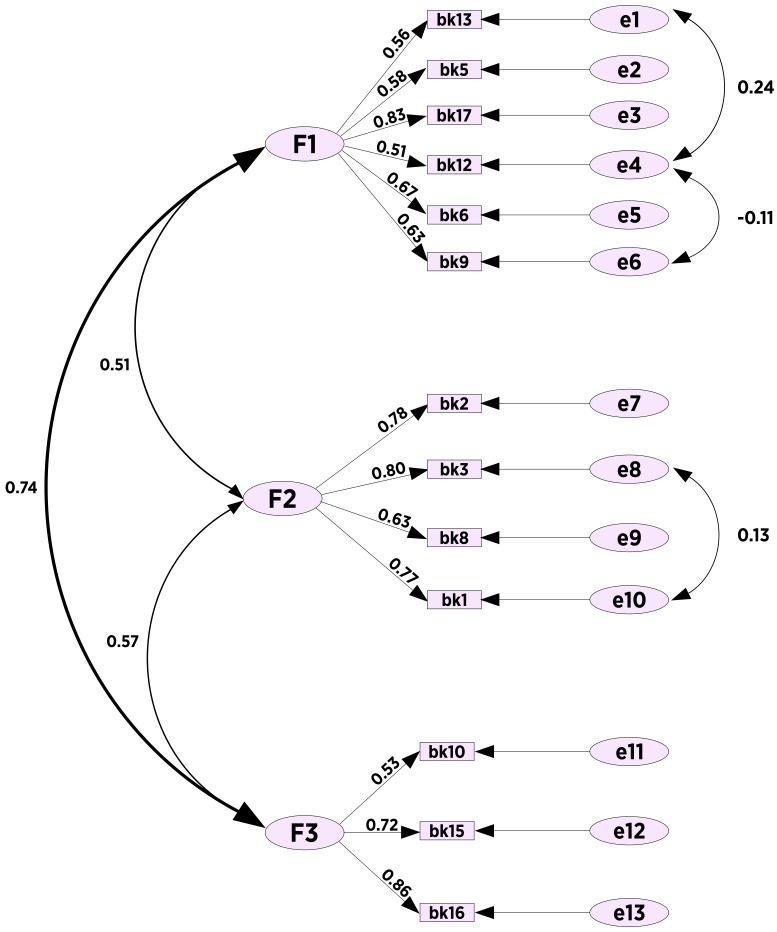
**Fear of failure scale path analysis**.

After the four mentioned items were removed, CMIN/df, GFI, AGFI, CFI and RMSEA were determined as 2.991, 0.897, 0.849, 0.604 and 0.085, respectively. In line with the recommendations of the correction indexes, the error covariances of some items were correlated. Accordingly, the analyses were performed after correlating the error covariances of items 1, 4 and 4, 6 in the first factor and items 8 and 10 in the second factor. Accordingly, CMIN/df, GFI, AGFI, CFI and RMSEA were found as 2.727, 0.911, 0.863, 0.673, and 0.079, respectively. The χ^2^ value in the first statistical process was CMIN = 366.4, Degree of Freedom (DF) = 116; however, with the corrections made, the χ^2^ value decreased to CMIN = 160.9, DF = 59. The standardized factor loadings in the model are statistically significant. These values vary between 0.52 and 0.83 for the first factor (emotions), 0.68 and 0.85 for the second factor (thoughts), and 0.55 and 0.84 for the third factor (physical symptoms). It was determined that these values showed a goodness of fit index [45] (Fig. 1).

The validity of similar scales was examined using the social anxiety scale. As fear of failure has been associated with social anxiety in the relevant literature, the relationship between the fear of failure scale and the social anxiety scale was examined. The analysis revealed a statistically significant positive correlation between the fear of failure scale and the social anxiety scale (r = 0.515, *p* = 0.001, and *p* < 0.001).

## 4. Discussion

Fear of failure is the relationship between the individual and the outcomes that will be obtained as a result of his/her failure [2]. It should be emphasized that it plays a very important role especially in the identity acquisition process during adolescence. The adolescent actually lays the foundations of most behaviors that he/she will exhibit in the future as a result of the habits and experiences acquired during this period.

The Performance Failure Assessment Inventory (PFAI), first developed by Conroy [2] to measure fear of failure, was adapted into Turkish by Kahraman and Sungur in 2016 [28]. The researchers confirmed the 5-factor structure of the 25 items in Conroy’s PFAI short version. When the items of this scale, which was adapted to Turkish with middle school students, are examined, it is seen that it mostly measures cognitive elements. The reason why Kandemir scale was used instead of Conroy (2001) [2] in this study is that it is shorter (17 items) and that its items include emotional and physical symptoms along with cognitive variables [2, 32]. For this reason, it was assumed that it would be important to introduce a measurement tool to the literature that measures fear of failure in adolescents in a variety of dimensions (physical, emotional, thoughts).

This study demonstrates the validity and reliability of the Fear of Failure Scale developed by Kandemir [32] among adolescents. An examination of the estimate values shows that the Fear of Failure Scale - Adolescent Form (FFS-A) is fit for a three-factor analysis. However, since the estimate values of items 4, 7, 11 and 14 were below 0.70 according to the item analysis results, they were removed from the scale. As a result, the FFS-A form consisted of 13 items with three sub-dimensions. The content of this form is related to thoughts, emotions and physical symptoms related to fear of failure. The internal consistency coefficients of the sub-dimensions of the FFS-A form related to thoughts, emotions and physical symptoms were determined as 0.84, 0.81 and 0.72, respectively. In the item analysis, it was seen that the item-total score correlations were between 0.326 and 0.679. While the specified value shows a sufficient level of correlation, it has been determined that the scale items have predictive and discriminatory power for total score. As a result, it is observed that the FFS-A form is reliable.

Anxiety felt about being evaluated or exams can cause students to perform lower, which results in academic failure. Anxiety experienced especially about exams can be explained through failure obsessions and affectivity components. Failure obsessions is the individual’s negative evaluation of himself/herself and his/her performance expectations, and comparing the situations related to the results of failure with others. On the other hand, affectivity refers to tension and emotional and physiological processes resulting from automatic action. In other words, students develop some detrimental negative thoughts during an evaluation process as a result of which they can experience failure [46].

Therefore, it becomes important for a person to be able to cope with the problems that will arise due to the social and emotional difficulties they may suffer from. These three factors in the scale provide a useful measurement tool to determine whether students’ symptoms are more intense in terms of physical, emotional or thought dimensions, or which of these are experienced together.

As a result of the explanatory factor analysis, it was seen that the scale had a three-factor structure explaining 56.55% of the variance. In the factor analysis conducted to examine the construct validity of the scale, it was observed that it has a three-factor structure. Accordingly, the first sub-dimension of the scale consisting of 6 items has loadings from items 5, 6, 9, 12, 13, and 17, the second sub-dimension consisting of 8 items has loadings from the second component consisting of items 1, 2, 3, and 8, and the third sub-dimension consisting of 3 items has loadings from the third component consisting of items 10, 15, and 16. When the scale items were examined in detail, it was determined that the first factor, the thoughts sub-dimension, was related to negative thoughts about the individual being perceived negatively and inadequately by important people or others in their environment. It was also found out that the thought patterns that individuals develop about not being able to focus on the course/exam due to their fears, being unsuccessful, or not being able to start to study play an important role. The second factor, emotions, includes expressions representing fear and intense anxiety. Finally, it was determined that the third factor examined in the scale was related to physical symptoms and the items were related to sleep problems, tension, and restlessness.

In the correlation analysis of the FFS-A and the adolescent social anxiety scale, the coefficient was calculated as r = 0.515 (*p* < 0.001). This value shows that there is a positive and moderate correlation between the two measurement tools. As the scores obtained from the SAS-A by the adolescents participating in the study increase, so does the total score of the FFS-A and the scores they receive from the sub-dimensions. In other words, the scores from the adolescent social anxiety scale increases with the scores obtained from the adolescent form of fear of failure. Therefore, a positive correlation was determined between both scales. A study conducted with secondary school students revealed findings that fear of academic failure was witnessed in children with social anxiety [47].

## 5. Conclusions

It is known that the new and special conditions that students are in during the pandemic period increase their anxiety levels [48]. It is thought that the uncertainty created by the measures taken during the pandemic period and the distance education process increased students’ anxiety and therefore may have cause them to perceive fear of failure more intensely.

In order to control the fear of failure seen in individuals during adolescence and to help increase their academic success, it was thought that a scale that can be used in school-based guidance and psycho-education practices or individual counseling is needed. It is also believed that these three factors of the scale can help the practitioner in determining whether the students’ symptoms are more intense in terms of physical, emotional or thought dimensions. These findings will shed light on which areas the practitioner should intervene in when conducting studies on fear of failure.

Fear of failure is among the problems that students frequently experience due to direct or indirect reasons. Especially the lack of prevalence studies on fear of failure in secondary school students in the TRNC can be seen as the proof of the necessity of this scale. Therefore, it is thought that this study will contribute to psychological counselors working in secondary schools identifying students with fear of failure and intervening as soon as practicable.

Both the construct validity and concurrent validity analysis results of the scale reveal that it has validity and reliability. Although the CFI score is below acceptable range, this may be because answers were collected through an online platform during the pandemic period from adolescents who experience the “abstract operational” stage of cognitive development, as suggested by Piaget. In this stage, the ability to think abstractly is still under construction [49]. Problems like being in pandemic period, experiencing pandemic-related restrictions such as school shut-downs, being isolated from friends, and challenges posed by adaptation to online education may have caused confusion among students about the survey questions. Considering that many of the students participating in this study may have taken an online survey for the first time, it is possible that the students may have perceived the survey as an exam. Therefore, we believe that face-to-face applications by researchers will allow for more detailed evaluations.

### Limitations of the Study

The data for this study were collected online during the 2021–2022 academic year, when there was a limited and gradual transition to face-to-face education following the period characterized with extraordinary restrictions due to the pandemic. One of the most important limitations of this application is that data could not be collected face to face. In the Turkish Republic of Northern Cyprus, schools were first to be intervened within the scope of pandemic measures. For this reason, the research questions were delivered to students receiving distance education via online platforms.

It is known that online research has its advantages as well as disadvantages. For example, reaching more people at a lower cost and faster is considered to be the biggest advantage of online research from the perspective of the practitioner. However, on the other hand, Wright states that the researcher may encounter a major problem when it comes to sampling: the demographic variables participants of the survey and/or tests are not observable [50]. On the other hand, the characteristics of the people who respond to the survey are questionable [51]. In this study, the researchers were aware of the disadvantages of conducting online research. On the other hand, since it was known that another application method would not be suitable in terms of time and cost due to the restrictions during the pandemic process, the data were collected considering these possible risks. For this reason, our suggestion for future research is to collect the data face-to-face and apply it to different cultures and age groups.

## Availability of Data and Materials

The data presented in this study are available on request from the corresponding author. The data are not publicly available due to privacy.
